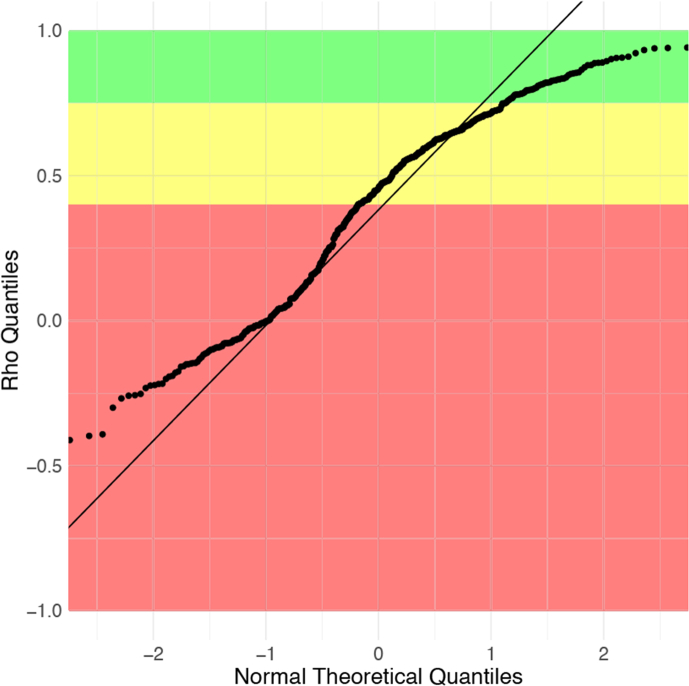# Correlation between Olink and SomaScan proteomics platforms in adults with a Fontan circulation

**DOI:** 10.1016/j.ijcchd.2025.100584

**Published:** 2025-04-15

**Authors:** Ismael Z. Assi, Michael J. Landzberg, Kristian C. Becker, David Renaud, Fernando Baraona Reyes, David M. Leone, Mark Benson, Miriam Michel, Robert E. Gerszten, Alexander R. Opotowsky

**Affiliations:** a Heart Institute, Department of Pediatrics, Cincinnati Children's Hospital, University of Cincinnati College of Medicine, Cincinnati, OH, USA; b Department of Cardiology, Boston Children's Hospital, Boston, MA, USA; c Department of Medicine, Brigham and Women's Hospital, Boston, MA, USA; d Harvard Medical School, Boston, MA, USA; e Fundamental and Biomedical Sciences, Paris-Cité University, Paris, France; f Health Sciences Faculty, Universidad Europea Miguel de Cervantes, Valladolid, Spain; g CardioVascular Institute, Beth Israel Deaconess Medical Center, Boston, MA, USA; h Division of Cardiovascular Medicine, Beth Israel Deaconess Medical Center, Boston, MA, USA; i Department of Child and Adolescent Health, Division of Pediatrics III — Cardiology, Pulmonology, Allergology and Cystic Fibrosis, Medical University of Innsbruck, Innsbruck, Austria

**Keywords:** Precision medicine, Plasma proteomics, Olink, SomaScan, Fontan circulation, Adult congenital heart disease

## Abstract

**Background:**

High-throughput proteomics platforms using aptamers (SomaScan) or proximity extension assay (Olink) provide novel opportunities for improving diagnostic and risk stratification tools in cardiovascular diseases, including understudied congenital heart diseases. The correlation between these proteomics approaches has not yet been studied among individuals with a Fontan circulation.

**Objective:**

The correlation of plasma protein measurements between SomaScan and Olink platforms was evaluated in adults with a Fontan circulation.

**Methods:**

We measured 491 proteins in plasma of 71 adults with a Fontan circulation using Olink and SomaScan. Missing Olink measurements (0.13%, 47/34,861) were imputed using non-parametric imputation. Spearman's rank correlation coefficient for absolute values of protein expression between platforms was calculated. Protein correlation frequencies were compared to 3 cohorts reported in the literature using Pearson's Chi-squared test of independence.

**Results:**

Overall, protein correlations between Olink and SomaScan measurements were moderately strong for most proteins, (rho > 0.4 for 57.2%), but with substantial variability (median correlation = 0.457, IQR = 0.538). The distribution of protein correlations was qualitatively similar to published literature in non-Fontan cohorts. Both Olink and SomaScan identified proteins with sex-based differences; both identified differences in myostatin and leptin, but each identified additional nonoverlapping sexually dimorphic proteins (n = 14 Olink, n = 5 SomaScan).

**Conclusions:**

In adults with a Fontan circulation, correlations between plasma proteins measured by Olink and SomaScan varied widely, approximately in line with prior reports in other populations. While these tools may be uniquely useful to generate hypotheses, specifically regarding potential molecular mechanisms, more definitive inference requires independent validation.

## Highlights

• Plasma proteomics platforms may provide insights into the pathophysiology of the Fontan circulation, a state associated with adverse outcomes and poorly understood mechanisms.
• We studied the correlation between the Olink and SomaScan proteomic platforms, describing moderate correlation; the results were qualitatively similar to other reported cohorts of biventricular patients.
• This suggests that the validity of these platforms in the Fontan circulation may be similar to that seen in cohorts with biventricular patients.

## Graphical abstract

In this study, we performed high throughput plasma proteomics profiling using Olink and SomaScan platforms on a cohort of patients with single ventricle Fontan circulation. Key results for measurement correlations between Olink and SomaScan, a comparison of protein correlation frequency to literature reports, and phenotypic validation of differential expression of sex proteins are summarized. Illustration created via BioRender (BioRender, Toronto, Canada, biorender.com). Abbreviations: Rho = Spearman's rank correlation coefficient; NHS = Nurses' Health Study [4]; ARIC = Atherosclerosis Risk in Communities [7]; HCM = hypertrophic cardiomyopathy patients undergoing septal ablation [6].

**Figure:**
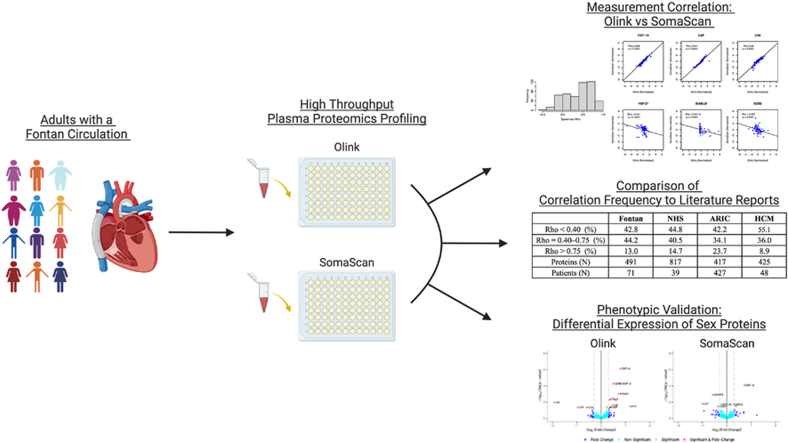

## Teaser

*Tweet Summarizing this Article:* @CincyChildrens @BostonChildrens @BIDMCHealth researchers have investigated how well high-throughput proteomics @SomaLogic @OlinkProteomics correlate in Fontan patients. These platforms may provide unique opportunities for developing cardiovascular risk stratification models.

## Abbreviations:

Olink: Olink proteomics platform
SomaScan: SomaLogic proteomics platform
ELISA: enzyme-linked immunosorbent assay
IRB: institutional review board
NYHA: New York Heart Association
PEA: proximity extension assay
qPCR: quantitative polymerase chain reaction
SOMAmer: slow off-rate modified aptamer
RFU: relative fluorescence unit
Rho: Spearman's rank correlation coefficient
Log_2_FC: log_2_ fold change
NHS: Nurses' Health Study
ARIC: Atherosclerosis Risk in Communities Study
HCM: cohort of hypertrophic cardiomyopathy patients undergoing septal ablation
SCAPIS: Swedish CArdioPulmonary bioImage Study
AV: atrioventricular
BMI: body mass index

**Protein Abbreviations**
NT-proBNP: N-terminal prohormone of brain natriuretic peptide
GDF15: Growth differentiation factor 15
ST2: Suppression of tumorigenicity 2
CysC: Cystatin C
FGF-19: Fibroblast Growth Factor 19
LEP: Leptin
CA6: Carbonic anhydrase 6
HSP 27: Heat shock protein 27
DIABLO: Diablo homolog i.e. second mitochondria-derived activator of caspases
SOD2: Superoxide dismutase 2
GH: Growth hormone
GDF-8: Myostatin
CD38: Cyclic ADP ribose hydrolase
MMP-3: Matrix metalloproteinase-3
PROK1: Prokineticin 1
RELT: Tumor necrosis factor receptor superfamily member 19L
MB: Myoglobin
BCAM: Basal cell adhesion molecule precursor
PPY: pancreatic polypeptide
IGFBP3: Insulin-like growth factor-binding protein 3
CD84: SLAM family member 5
CXCL16: C-X-C motif chemokine ligand 16
ICAM-2: Intercellular adhesion molecule 2
ANGPTL4: Angiopoietin-like 4

## 1 Introduction

Two proteomics platforms, one from Olink Proteomics AB (“Olink”, Olink Target 96 Panel, Uppsala, Sweden) and another from SomaLogic (“SomaScan”, SOMAscan HTS Assay 1.3K, Boulder, United States) have recently made advances to enable the high-throughput relative quantification of thousands of human plasma proteins. Several studies have investigated the accuracy of both platforms against absolute quantification techniques such as mass-spectrometry and ELISA measurements in blood plasma and cerebral spinal fluid, reporting correlations that were modest and with significant variability depending on the proteins measured [[1], [2], [3]]. Other studies have performed direct pairwise comparisons of Olink and SomaScan, reporting moderate correlations between the platforms [[2], [3], [4], [5], [6], [7]].

Given the adverse health outcomes that adults with a Fontan circulation face [[8], [9], [10], [11]] and with the uncertainty about whether diagnostics used in acquired heart disease are appropriate to apply to those born with congenital heart disease (CHD) [12], the use of high-throughput proteomics platforms provides unique opportunities for the development of CHD-specific biomarkers for diagnostic and risk stratification models. This is especially relevant given that the Fontan circulation is associated with a unique physiologic state in which passive venous return and cardiac remodeling affect end organ systems leading to complications such as heart failure, arrhythmia, plastic bronchitis, protein losing enteropathy, hepatocellular carcinoma, and chronic kidney disease [[13], [14], [15], [16]]. Furthermore, given that clinicians face difficulties foreseeing major adverse events in Fontan patients [17,18], investigators have become interested into whether molecular signals may give insights into the mechanisms of Fontan-associated pathophysiology [19,20].

However, it is unclear whether previous studies into Olink and SomaScan platforms are generalizable to patients with a Fontan circulation due to their unique physiology and medical management. This uncertainty is particularly relevant given that patients with a Fontan palliation are often on anticoagulation therapy, and several studies have raised concerns about artifacts associated with the SomaScan platform in patients receiving heparin [21]. Therefore, in this study, we aimed to 1) evaluate the correlation between proteins assessed by Olink and SomaScan and a select number of validated clinical assays, 2) evaluate whether intraplatform correlations in adults with a Fontan circulation are consistent with previous studies of patients with a biventricular circulation, and 3) assess the face validity of each platform in this Fontan cohort by evaluating whether proteins with significant sexual dimorphism align with findings from prior reports (**Central Illustration**).

## 2 Methods

### 2.1 Participant enrollment

We studied adults (>18 years old) with a Fontan circulation seen at Boston Children's or Brigham and Women's Hospitals between 2012 and 2017 as part of the Boston Adult Congenital Heart Disease Biobank [22]. The study was approved by the Boston Children's Hospital Institutional Review Board. IRB approval was extended to patients enrolled at the Brigham and Women's Hospital via a formal reliance agreement. All work was carried out in accordance with the World Medical Association Declaration of Helsinki. Written informed consent was acquired from all study participants.

Demographic and clinical data were acquired on the date of biospecimen collection. Clinical data extracted from health records included resting arterial oxygen saturation, New York Heart Association (NYHA) functional class (I vs. ≥II), and current anticoagulant usage.

### 2.2 Biospecimen collection

Peripherally drawn blood plasma from non-fasting patients was collected in an ethylenediaminetetraacetic acid tube and centrifuged for 10 min at 1300g and 4 °C. Aliquoted samples were stored at −80 °C. Samples were collected between 2012 and 2017. SomaScan measurements (June 2017) were performed 1.5 years earlier than the Olink measurements (December 2018), using different aliquots collected as part of the same blood draw. There were no freeze-thaw cycles prior to the current analysis.

### 2.3 Proteomics profiling

The Gerszten Lab at Beth Israel Deaconess Medical Center performed the proteomics examinations using two different commercially available proteomics platforms for 71 participants (Olink platform, comprising 975 proteins; SomaScan platform, comprising 1265 proteins) (Supplemental Methods 1).

The Olink platform (Olink Target 96 Panel) leverages Proximity Extension Assay (PEA) technology coupled with qPCR to quantify protein expression. We selected to measure proteins from 11 previously established expression panels in our analysis: Cardiometabolic, Cardiovascular II, Cardiovascular III, Cell Regulation, Development, Immune Response, Inflammation, Metabolism, Neurology, Oncology II, and Organ Damage. For each target protein within each expression panel, complementary oligonucleotide-coupled antibody probes bind to unique epitopes, which subsequently enable hybridization of the oligonucleotides. The hybridized oligonucleotides form a template that is extended by the addition of DNA polymerase and quantified by qPCR [23]. The PEA-qPCR readout is presented as a log_2_-scaled value which is proportional to protein expression. Each protein is then assigned a limit of detection estimated using negative controls. Measurement values below the limit of detection were included in this analysis.

The SomaScan platform (SOMAscan HTS Assay 1.3K) is an orthogonal protein quantification strategy which uses the SOMAmer (Slow Off-rate Modified Aptamer) proprietary protein-capture technique. Streptavidin-bound SOMAmer reagents are used to capture proteins of interest which are then denatured. The denatured proteins are hybridized to complementary fluorophore-labelled nucleic acid probes. The final readout for each probe is an arbitrary relative fluorescence unit (RFU) which is proportional to protein expression. To standardize measurement scales between SomaScan and Olink, SomaScan RFU were log_2_-scaled.

### 2.4 Clinical biomarker assays

N-terminal prohormone of brain natriuretic peptide (NT-proBNP), cystatin C (CysC), growth differentiation factor 15 (GDF15), and suppression of tumorigenicity 2 (ST2) were measured from different aliquots of stored plasma that had been collected as part of the same blood draw during which the blood had been collected for the proteomics analysis. NT-proBNP (Elecsys ProBNP II Immunoassay, Catalog Number 04842464160) and CysC (Tina-quant Cystatin C, Catalog Number 04975723190) were measured from frozen plasma samples using biomarker specific immunoassays with a Cobas 8000 analyzer (Roche Diagnostics, Indianapolis, IN). GDF15 (Catalog Number DGD150) and ST2 (Catalog Number DST200) were measured from absorbance readings of a microplate reader set to 450 nm (Quantikine ELISA, R & D Systems Minneapolis, MN). For NT-proBNP, CysC, GDF-15, and ST2 the lower limits of detection were**:** 5 pg/mL, 0.4 mg/L, 2.0 pg/mL, and 5.1 pg/mL respectively with sample values below these limits reported as half of the lower limit value. These assays were performed at the Clinical and Epidemiologic Laboratory (CERLab) within the Department of Laboratory Medicine at Boston Children's Hospital, certified by the Clinical Laboratory Improvement Amendments (CLIA) and the College of American Pathologists (CAP).

### 2.5 Data imputation

UniProt ID (https://www.uniprot.org/) [24] was used to identify overlapping proteins between platforms. SomaScan measurements were available for all proteins in all samples, whereas 47 out of the 34,861 (0.13%) measurements were unavailable for the Olink dataset. Missing Olink measurements were imputed using the MissForest non-parametric model [25] with 100 trees in each forest until the estimated error was less than 0.10. Data imputation and all subsequent statistical analysis was performed using R version 4.4.0 (https://www.r-project.org/).

### 2.6 Pairwise protein analysis

Spearman's rank correlation coefficient (rho) was estimated between Olink and SomaScan measurements with corresponding clinical assays of CysC, GDF15, NT-proBNP, and ST2. Rho was also estimated for every overlapping protein between Olink and SomaScan. Normality of the rho distribution between Olink and SomaScan was evaluated using a one sample Kolmogorov-Smirnov test. Frequencies of protein correlation between Olink and SomaScan were compared to frequencies reported in the Nurses' Health Study (NHS) [4], Atherosclerosis Risk in Communities (ARIC) study [7], and a cohort of hypertrophic cardiomyopathy patients undergoing septal ablation (HCM) [6] using Pearson's Chi-squared test of independence. Post-hoc analysis was performed using pairwise chi-squared tests corrected using the Benjamini-Hochberg procedure [26].

### 2.7 Analysis of sexually dimorphic proteins

Log_2_ fold change (log_2_FC) was calculated by subtracting the mean of log_2_-scaled protein measurement between males and females for both Olink and SomaScan. Test statistics were computed using a two-sided Welch's *t*-test. P-values were adjusted using the Benjamini-Hochberg procedure. Spearman correlation was then used to compare the log_2_FC of proteins in the Fontan cohort to values reported in the Swedish CArdioPulmonary bioImage Study (SCAPIS) [27].

## 3 Results

### 3.1 Cohort overview

A total of 71 participants with a Fontan circulation had 975 proteins profiled via Olink and 1265 proteins profiled via SomaScan. Pairwise measurements were available for 491 proteins. The average age was 29 ± 7 years, 82% were non-Hispanic white, and 48% were female (Table 1).

**Table 1:** Participant demographic and clinical characteristics.

**Variable**	
Participants	71
Age, years	29.1 ± 7.2
Height, cm	165.8 ± 10.1
Weight, kg	68.6 ± 14.6
BMI, kg/m^2^	24.8 ± 4.0
O_2_ saturation, %	93.6 ± 4.3
**Sex**	
Male	37 (52.1)
Female	34 (47.9)
**Ethnicity**	
White non-Hispanic	58 (81.7)
Black	1 (1.4)
White Hispanic	3 (4.2)
Asian	2 (2.8)
Other	1 (1.4)
Unknown	6 (8.5)
**NYHA functional class**	
I	47 (66.2)
II-III	23 (32.4)
Unknown	1 (1.4)
**Anticoagulation**	
Aspirin	41 (57.7)
Warfarin	23 (32.4)
Other/Unknown	2 (2.8)
None	5 (7)
**Congenital diagnosis**	
Hypoplastic left heart syndrome	11 (15.5)
Tricuspid atresia	16 (22.5)
Double inlet left ventricle	14 (19.7)
Unbalanced AV septal defect	2 (2.8)
Other	28 (39.4)

Legend: Continuous variables are presented as mean ± standard deviation. Categorical variables are presented as counts and percentage of total.

Abbreviations: AV = atrioventricular; BMI = body mass index; NYHA = New York Heart Association.

### 3.2 Correlation between clinical assays and proteomics platforms

**Fig. 1:**
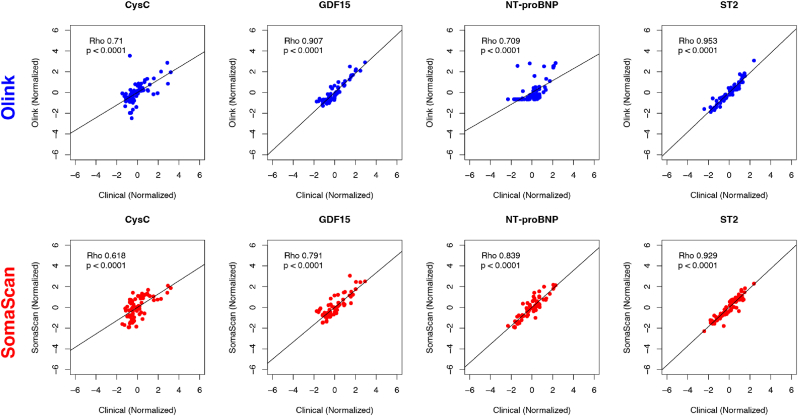
Legend: Standardized scatterplots of log_2_-scaled protein measurements between clinical assays and Olink (blue) or SomaScan (red). Abbreviations: Rho = Spearman's rank correlation coefficient; CysC = Cystatin C; GDF15 = Growth differentiation factor 15; NT-proBNP = N-terminal prohormone of brain natriuretic peptide; ST2 = Suppression of tumorigenicity 2.

Fig. 1 displays the correlation between the 4 proteins for which there were clinical assays and Olink or SomaScan measurements. All correlations displayed in Fig. 1 were significant (p < 0.0001). In general, Olink demonstrated a stronger correlation rho with clinical assays than SomaScan (CysC = 0.710 vs 0.618, GDF15 = 0.907 vs 0.791, NT-proBNP = 0.709 vs 0.839, and ST2 = 0.953 vs 0.929).

### 3.3 Correlation between olink and SomaScan platforms

The estimated rho between Olink and SomaScan measurements were not normally distributed (Kolmogorov–Smirnov test, D = 0.398, p < 0.0001 and Supplemental Fig. 1). The median correlation coefficient was 0.457; the 25th percentile was 0.113 and 75th percentile was 0.651. Of the 11 Olink panels measured, the Development panel demonstrated the strongest correlations (median = 0.651, Q1-Q3 = 0.384–0.724) and the Cell Regulation panel demonstrated the weakest correlations (median = 0.133, −0.037-0.481) (Table 2).

**Table 2:**
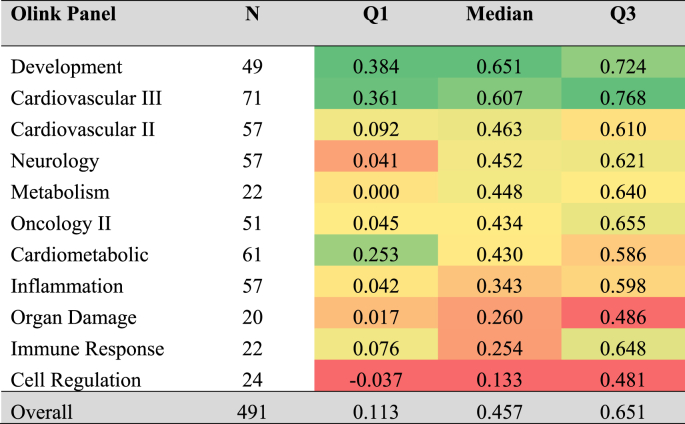
Q1, median, and Q3 of Spearman's rank correlation coefficient estimated between Olink and SomaScan protein measurements.

**Fig. 2:**
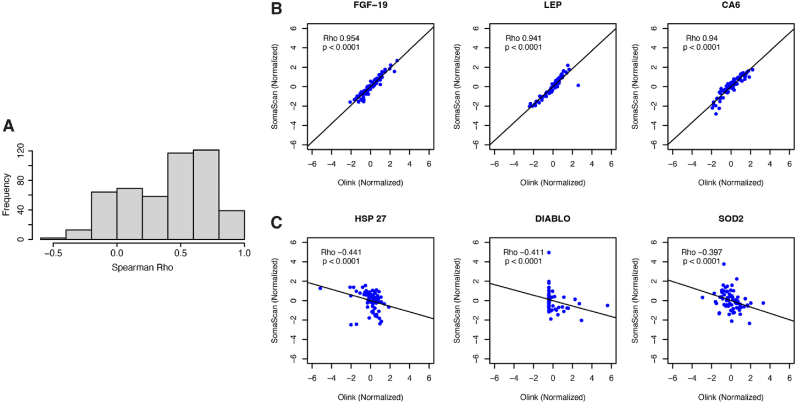
Legend: **A)** Histogram of rho between Olink and SomaScan for overlapping proteins (N = 491). Standardized scatterplots of log_2_-scaled protein measurements for the 3 proteins with the **B)** most positive and **C)** most negative correlations. Abbreviations: Rho = Spearman's rank correlation coefficient; FGF-19 = Fibroblast Growth Factor 19; LEP = Leptin; CA6 = Carbonic anhydrase 6; HSP 27 = Heat shock protein 27; DIABLO = Diablo homolog i.e. second mitochondria-derived activator of caspases; SOD2 = Superoxide dismutase 2.

The distribution of rho is depicted in Fig. 2A. (see detailed list in Supplemental Table 1). Negative correlations were observed for 79 proteins. Fig. 2B displays the 3 proteins (fibroblast growth factor 19 = FGF-19, leptin = LEP, carbonic anhydrase VI = CA6) with the most positive correlation, whereas Fig. 2C displays the 3 proteins (heat shock protein 27 = HSP 27, diablo homolog = DIABLO, superoxide dismutase 2 = SOD2) with the most negative correlation. All correlations displayed in Fig. 2B and C were significant (p < 0.0001). Of note, 47 DIABLO measurements were below the Olink limit of detection.

The distribution of correlations in the Fontan cohort was then compared to results reported in the NHS, ARIC, and HCM studies [4,6,7] (Table 3) by stratifying rho into 3 groups (rho < 0.4, rho = 0.4–0.75, and rho > 0.75). The distribution of counts across cohorts was qualitatively similar, however a significant difference was detected (X-squared = 50.8, df = 6, p = 3.3e-9). Pairwise chi-squared comparisons corrected using the Benjamini-Hochberg procedure failed to detect a difference between Fontan and NHS cohorts (adjusted p = 0.39), but differences were detected for the remaining comparisons (adjusted p were Fontan: ARIC = 3.9e-5, Fontan: HCM = 7.8e-4, NHS: ARIC = 3.1e-4, NHS: HCM = 6.1e-4, and ARIC: HCM = 1.8e-8).

**Table 3:** Comparison of Olink-SomaScan correlation frequencies between Fontan, NHS, ARIC, and HCM cohorts.

	Fontan	NHS	ARIC	HCM
Rho <0.40 (%)	42.8	44.8	42.2	55.1
Rho = 0.40–0.75 (%)	44.2	40.5	34.1	36.0
Rho >0.75 (%)	13.0	14.7	23.7	8.9
Proteins (N)	491	817	417	425
Participants (N)	71	39	427	10

Legend: The distribution of correlation frequencies was qualitatively similar across cohorts that had varying exclusion criteria and different proteins measured. Pearson's Chi-squared test indicated a significant difference between cohorts (X-squared = 50.8, df = 6, p-value = 3.3e-9). Pairwise chi-squared comparisons corrected using the Benjamini-Hochberg procedure failed to detect a difference between Fontan and NHS cohorts (adjusted p = 0.39) but detected differences for the remaining comparisons (all adjusted p < 0.005).

Abbreviations: Rho = Spearman's rank correlation coefficient; NHS = Nurses' Health Study [4]; ARIC = Atherosclerosis Risk in Communities [7]; HCM = hypertrophic cardiomyopathy patients undergoing septal ablation [6].

### 3.4 Differentially expressed sex proteins

**Fig. 3:**
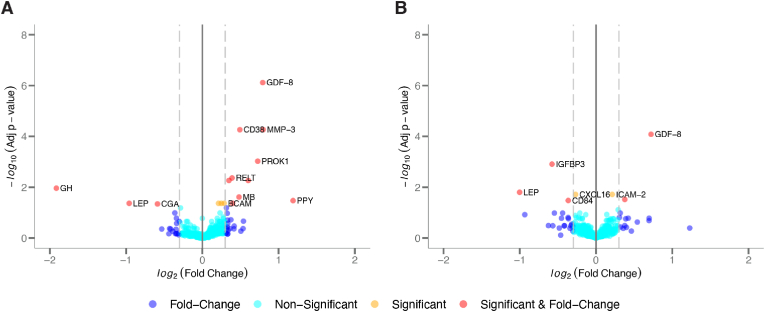
Legend: Volcano plots of protein male-female log_2_ fold change for **A)** Olink and **B)** SomaScan. For each platform, either an adjusted p-value less than 0.05 or fold change greater than 0.3 was considered a meaningful difference between male and female. Abbreviations: GH = Growth hormone; LEP = Leptin; GDF-8 = Myostatin; CD38 = cyclic ADP ribose hydrolase; MMP-3 = Matrix metalloproteinase-3; PROK1 = Prokineticin 1; RELT = Tumor necrosis factor receptor superfamily member 19L; MB = Myoglobin; BCAM = Basal cell adhesion molecule precursor; PPY = pancreatic polypeptide; IGFBP3 = Insulin-like growth factor-binding protein 3; CD84 = SLAM family member 5; CXCL16 = C-X-C motif chemokine ligand 16; ICAM-2 = Intercellular adhesion molecule 2.

Fig. 3 displays volcano plots of differentially expressed sex proteins, comparing male (N = 37) and female (N = 34) participants for **A)** Olink and **B)** SomaScan platforms. Myostatin (GDF8) had the most significant adjusted p-value for both Olink (log_2_FC = 0.792, adjusted p = 7.6e-7) and SomaScan (log_2_FC = 0.722, adjusted p = 1.7e-7). Olink had 16 proteins that demonstrated a significant sexual dimorphism (FDR adjusted p < 0.05) whereas 7 SomaScan proteins were significant (see full protein list in Supplemental Table 2 **for Olink** and Supplemental Table 3 **for SomaScan**). For Olink, the most dominantly expressed protein fold change between males and females were observed for pancreatic polypeptide (PPY, log_2_FC = 1.190, adjusted p = 0.033) and growth hormone (GH, log_2_FC = −1.915, adjusted p = 0.011). The most dominantly expressed protein fold change for SomaScan were sialic acid-binding Ig-like lectin 9 (Siglec-9, log_2_FC = 1.229, adjusted p = 0.401) and leptin (LEP, log_2_FC = −1.003, adjusted p = 0.016).

**Fig. 4:**
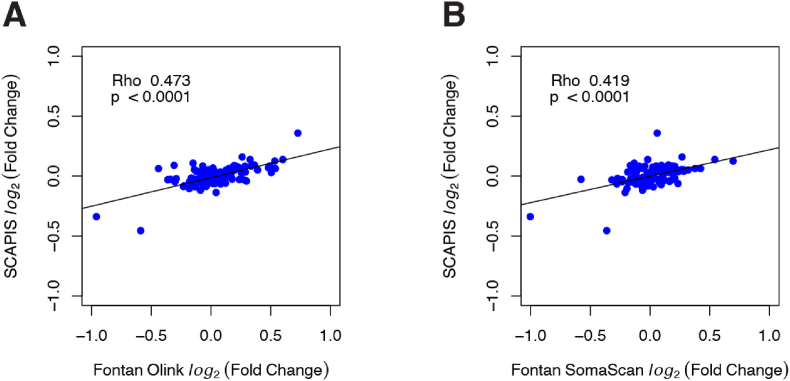
Legend: Scatterplots of log_2_ fold change in male-female protein expression (N = 123) between results reported in the SCAPIS study [27] and **A)** Fontan Olink or **B)** Fontan SomaScan. Abbreviations: SCAPIS = Swedish CArdioPulmonary bioImage Study.

Log_2_FC between males and females in the Fontan cohort were then compared to results reported in the SCAPIS study [27]. Fontan-Olink:SCAPIS had a correlation of 0.473 (p < 0.0001) whereas Fontan-SomaScan:SCAPIS had a correlation of 0.419 (p < 0.0001) (Fig. 4). The detailed list of male-female log_2_FC across cohorts is provided in Supplemental Table 4.

## 4 Discussion

This study investigated the relationship between overlapping proteins measured by two different commercially available proteomics platform in adult patients with a Fontan circulation. Our results suggest that in plasma samples collected from non-fasting ambulatory adults with a Fontan circulation, circulating protein concentrations assessed by Olink and SomaScan correlated well with clinical assays of cystatin C, growth differentiation factor 15, N-terminal prohormone of brain natriuretic peptide, and suppression of tumorigenicity 2 (all rho were greater than 0.6). Furthermore, when comparing Olink and SomaScan directly, the protein concentrations correlated moderately well, but with significant variability between individual target proteins (median rho = 0.457, IQR = 0.538). Qualitatively, these correlations mirror those seen in the previously published literature, suggesting analyses utilizing these platforms in those with a Fontan circulation may be interpreted as they would for other disease populations and for the general population. However, the biological significance of the variability in correlation between platforms remains unclear, highlighting an important consideration for biomarker-based decision making. While the moderate agreement we observed between assays may be sufficient for broader observational research, clinical applications of proteomic findings will inevitably demand greater precision and consistency, as discrepancies in protein measurements could alter decision-making.

These findings can help inform future biomarker validation efforts by helping to focus investigation on proteins with poor correlation between platforms. Another finding that may guide future validation efforts is a deeper analysis into proteins that fall below the Olink limit of detection. For example, DIABLO, one of the proteins with the poorest correlation, had 47 out of 71 measurements below the Olink detection threshold, while SomaScan values ranged from 4.96 to −1.16 standard deviations from the mean (15.0–12.8 log_2_ RFU).

Irrespective of correlation between platforms, proteins of interest require validation against gold standard assays such as ELISA or mass spectrometry. This is required to interpret absolute values of protein measurements, establish reference values, and ensure batch-to-batch consistency of proteins measured via both platforms. Standardized sample collection and storage, instrument calibration, and comparing results against known protein references or control samples can also further help to ensure accuracy and biomarker validation.

Not only for proteomics assays there is still much to evaluate and validate, but that this may also hold true also for other -omics techniques. As noted in a recent study quantifying serum amino acids in congenital heart disease, a similar variability in amino acid concentrations measured using orthogonal platforms was observed [28]. This underlines the importance of positive controls in -omics studies and that the concerns raised in this study should also be taken into consideration when evaluating expression levels of alternative specimens and measurement techniques.

To assess the face validity of each platform in this Fontan cohort, we evaluated the differential expression of sex proteins. We found 16 plasma proteins with significant sexual dimorphism using Olink and 7 proteins using in SomaScan. Overall, the proteins differentially expressed agreed with prior literature on sex differences in circulating protein concentrations. Alternatively, two mutually significant proteins identified between the platforms were myostatin and leptin. Sexual dimorphism of myostatin and leptin has been previously reported [[29], [30], [31], [32]]. This analysis further suggests that sex-matched controls may be an important consideration in proteomics studies.

Lastly, we evaluated the correlation between male-female fold change in our Olink and SomaScan measurements against the results reported in the SCAPIS study [27]. Fontan-Olink had a stronger correlation (rho = 0.473) than Fontan-SomaScan (rho = 0.419). However, protein measurement in the SCAPIS study was performed using Olink PEA-qPCR which is a similar technique used in our Fontan-Olink cohort. Therefore, no definitive conclusions can be made regarding the observed correlation differences. We suggest that further studies are needed to validate differential protein concentrations based on participant phenotypes in both Olink and SomaScan platforms.

### 4.1 Limitations

These results must be interpreted in the context of the underlying study design and analytical approach. Most importantly, we did not validate a significant number of protein measurements using alternative platforms or gold-standard techniques such as ELISA assays or by mass spectrometry. Therefore, there are multiple possible interpretations of the weak correlation between Olink and SomaScan measurements. We also did not apply strategies to increase apparent measurement agreement between the platforms, such as excluding measurements below the limit of detection or excluding extreme outliers as has been done in some other studies [7]. We employed this strategy in order to reduce potential bias and increase the total number of pairwise comparisons, which we feel reflects the likely methods others will employ in future practice when comparing different proteomic platforms. While further curation of the proteins analyzed may increase the agreement between the platforms, this may be marginal given the consistency of the proteins with low congruency (rho <0.4) in our study and several others [4,6,7]. Furthermore, to maximize the number of pairwise comparisons in our study we imputed 47 missing measurements in the Olink dataset. While this could, in theory, impact correlation results, such an effect would be trivial given that only 0.14 % of measurements were imputed.

Furthermore, there were several differences among the cohorts compared in Table 3. The NHS cohort consisted of female nurses, the ARIC cohort included a diverse group of participants from the United States, and the HCM cohort comprised patients undergoing septal ablation for hypertrophic cardiomyopathy. Notably, the Fontan cohort had a lower mean age and BMI than the NHS and ARIC cohorts, whereas demographic data were unavailable for the HCM cohort (Supplemental Table 5). As such, a key limitation of the presented cohort comparison is that correlation results were not adjusted for potential confounding factors such as age, sex, BMI, and other relevant characteristics and comorbidities.

Another important consideration is that proteomic platforms are advancing, and the current study used prior versions of the Olink and SomaScan platforms. Improved sensitivity, specificity, or expanded protein coverage might influence comparisons between platforms. However, improvement is far from a certainty; a recent study using the updated Olink Explore 3072 and SomaScan Assay v4.1 platforms in a cohort of Chinese adults found a lower median correlation coefficient of 0.26 [33], than the median correlation of 0.46 observed in the current study. Ongoing investigation is needed to confirm whether the results outlined in this manuscript are applicable to the latest versions of the platforms.

Lastly, plasma samples were stored in −80 °C for approximately 3.5 years before performing the SomaScan assay and 5 years before performing the Olink assay. Recent studies have suggested that metabolites are stable for up to 7 years [34] in −80 °C and therefore it is not clear whether that the difference in storage time significantly impacted correlations between platforms. Furthermore, protein levels may not change uniformly over time in frozen samples which could potentially explain the differences in correlations observed between Olink panels. More studies are needed to investigate length of storage in −80 °C and its subsequent impact on SomaScan and Olink measurements.

### 4.2 Conclusions

Results of this study suggest that: (1) there is moderate correlation between Olink and SomaScan proteomics platforms in adults with a Fontan circulation, (2) the frequencies of correlation strength for proteins between platforms were qualitatively similar to cohorts that have been previously reported in the literature, and (3) there is some level of face validity of protein measurement, to the extent that there is consistency in identifying protein dimorphism between males and females by each platform. While these findings suggest that the use of SomaScan and Olink in patients with a Fontan circulation are a viable method for initial assessment and hypothesis generation, they also argue for caution in interpreting the results of proteomic analyses relying solely on such platforms given variable measurement performance and other aspects of uncertainty. While the ability to measure this volume of proteins simultaneously with a small biological sample is important for exploratory analysis may prove of great use to generate mechanistic hypotheses, key findings should be confirmed with validated assays before definitive conclusions are drawn.

## CRediT authorship contribution statement

**Ismael Z. Assi:** Writing – review & editing, Writing – original draft, Visualization, Validation, Supervision, Software, Project administration, Methodology, Investigation, Formal analysis, Data curation, Conceptualization. **Michael J. Landzberg:** Writing – review & editing, Methodology, Investigation, Data curation, Conceptualization. **Kristian C. Becker:** Writing – review & editing, Writing – original draft, Methodology, Investigation, Formal analysis, Data curation, Conceptualization. **David Renaud:** Writing – review & editing, Methodology, Investigation, Data curation, Conceptualization. **Fernando Baraona Reyes:** Writing – review & editing, Methodology, Investigation, Data curation, Conceptualization. **David M. Leone:** Writing – review & editing, Methodology, Investigation, Data curation, Conceptualization. **Mark Benson:** Writing – review & editing, Methodology, Investigation, Data curation, Conceptualization. **Miriam Michel:** Writing – review & editing, Methodology, Investigation, Data curation, Conceptualization. **Robert E. Gerszten:** Writing – review & editing, Resources, Methodology, Investigation, Data curation, Conceptualization. **Alexander R. Opotowsky:** Writing – review & editing, Writing – original draft, Validation, Supervision, Software, Resources, Project administration, Methodology, Investigation, Funding acquisition, Formal analysis, Data curation, Conceptualization.

## Data Statement

Protein-level correlations and fold changes are available as supplementary material.

## Disclosures

Professor Alexander (Sasha) Opotowsky is the one of the Twitter (X) Editors of the International Journal of Cardiology Congenital Heart Disease and played no role in the Journal's evaluation of the manuscript. The authors report no other relationships that could be construed as a conflict of interest.

## Declaration of generative AI

During the preparation of this work the authors used ChatGPT-4o (OpenAI, San Francisco, United States) for assistance with R code generation, data analysis techniques, and sentence transition structure. After using this service, the authors reviewed and edited the code and text as needed and take full responsibility for the content of the published article.

## Funding sources

This work was conducted with support from: the 10.13039/100000968American Heart Association Innovative Research Grant 17IRG33370023, 10.13039/100007299Harvard Catalyst/10.13039/100007299The Harvard Clinical and Translational Science Center (10.13039/100000097National Center for Research Resources and the 10.13039/100006108National Center for Advancing Translational Sciences, National Institutes of Health Award UL1 TR001102, 10.13039/100007229Harvard University, and its affiliated academic healthcare centers), the Heart Institute Research Core (10.13039/100031225HIRC) at Cincinnati Children's Hospital, and an investigator-initiated study research grant from 10.13039/100016545Roche Diagnostics (Indianapolis, IN). ARO is supported by the 10.13039/100000050National Heart, Lung, and Blood Institute (10.13039/100000050NHLBI) of the 10.13039/100000002National Institutes of Health, R01 HL151604. ARO, MJL, and FBR were supported by the Dunlevie Family Fund. ARO and DML were supported by the Heart Institute Research Core at 10.13039/100007172Cincinnati Children's Hospital, and the Samuel and Molly Kaplan Fund for Adult Congenital Heart Disease. IZA was supported by the Cardiovascular Medicine track of the 10.13039/100008102University of Cincinnati Medical Student Scholars Program. KCB was supported by a T32 Grant administered through the 10.13039/100008102University of Cincinnati and Cincinnati Children's Hospital (NIH/NIEHS T32ES010957). MDB was supported by K08HL145095.

## Declaration of competing interest

The authors declare the following financial interests/personal relationships which may be considered as potential competing interests: Alexander R. Opotowsky reports financial support was provided by 10.13039/100016545Roche Diagnostics. Alexander R. Opotowsky is the one of the Twitter (X) Editors of the International Journal of Cardiology Congenital Heart Disease and played no role in the Journal's evaluation of the manuscript. If there are other authors, they declare that they have no known competing financial interests or personal relationships that could have appeared to influence the work reported in this paper.

## Figures and Tables

**figs1:**