# Supplementary material for: Correlation between Olink and SomaScan proteomics platforms in adults with a Fontan circulation
Source: Int J Cardiol Congenit Heart Dis. 2025 Apr 15;20:100584. doi: 10.1016/j.ijcchd.2025.100584 (PMC12053979; doi:10.1016/j.ijcchd.2025.100584)
Supplement: Multimedia component 1 [file mmc1.docx]

**Correlation between Olink and SomaScan Proteomics Platforms in Adults with a Fontan Circulation**

Ismael Z. Assi, Michael J. Landzberg, Kristian C. Becker, David Renaud, Fernando Baraona Reyes, David M. Leone,

Mark Benson, Miriam Michel, Robert E. Gerszten, Alexander R. Opotowsky

**Supplemental Methods 1:** Sample Preparation for Olink

**Sample Type:** Plasma (ETDA/Citrated)

**Thawing and Aliquoting Sample Sets**

- Samples removed from a -80°C freezer and placed on a Rotator at 4°C
- Gentle mixing on a rotator until all plasma is thawed
- Solution pipetted up and down 2 or 3 times to assure complete mixing
- Thawed plasma immediately placed in an ice bucket with ice and water+ice slurry
- Tubes inspected for presence of debris and appropriate sample color
- Samples vortexed
- Supernatant withdrawn and placed in 96 well plate
- Store at -80°C till shipped.

**Sample volume required: 40 microliters per sample arranged in a 96 well plate, leaving A12-H12 empty for controls.** Following was the plate layout:

|  | **Plate Layout** | |  |  |  |  |  |  |  |  |  |  |
| --- | --- | --- | --- | --- | --- | --- | --- | --- | --- | --- | --- | --- |
|  | **1** | **2** | **3** | **4** | **5** | **6** | **7** | **8** | **9** | **10** | **11** | **12** |
|  | **Sample Plate** | | | | | | | | | | | |
| **A** | Sample0001 | Sample0009 | Sample0017 | Sample0025 | Sample0033 | Sample0041 | Sample0049 | Sample0057 | Sample0065 | Sample0073 | Sample0081 | **Control1** |
| **B** | Sample0002 | Sample0010 | Sample0018 | Sample0026 | Sample0034 | Sample0042 | Sample0050 | Sample0058 | Sample0066 | Sample0074 | Sample0082 | **Control2** |
| **C** | Sample0003 | Sample0011 | Sample0019 | Sample0027 | Sample0035 | Sample0043 | Sample0051 | Sample0059 | Sample0067 | Sample0075 | Sample0083 | **Ngt Cnt** |
| **D** | Sample0004 | Sample0012 | Sample0020 | Sample0028 | Sample0036 | Sample0044 | Sample0052 | Sample0060 | Sample0068 | Sample0076 | Sample0084 | **Ngt Cnt** |
| **E** | Sample0005 | Sample0013 | Sample0021 | Sample0029 | Sample0037 | Sample0045 | Sample0053 | Sample0061 | Sample0069 | Sample0077 | Sample0085 | **Ngt Cnt** |
| **F** | Sample0006 | Sample0014 | Sample0022 | Sample0030 | Sample0038 | Sample0046 | Sample0054 | Sample0062 | Sample0070 | Sample0078 | Sample0086 | **IPC** |
| **G** | Sample0007 | Sample0015 | Sample0023 | Sample0031 | Sample0039 | Sample0047 | Sample0055 | Sample0063 | Sample0071 | Sample0079 | Sample0087 | **IPC** |
| **H** | Sample0008 | Sample0016 | Sample0024 | Sample0032 | Sample0040 | Sample0048 | Sample0056 | Sample0064 | Sample0072 | Sample0080 | Sample0088 | **IPC** |
